# Ocular metastasis from breast carcinoma simulating anterior scleritis: a case report

**DOI:** 10.1186/s13256-017-1416-y

**Published:** 2017-08-28

**Authors:** Ritesh Kumar Shah, Samir Lamichhane

**Affiliations:** 1 Department of Ophthalmology, Childrens’ Hospital for Eye, ENT and Rehabilitation Services (CHEERS), Bhaktapur, Kathmandu, Nepal; 2 Department of Clinical Pharmacology, Maharajgunj Medical Campus, Institute of Medicine, Tribhuvan University, Kathmandu, Nepal

**Keywords:** Anterior scleritis, Breast cancer, Case report, Ocular metastasis, Subretinal mass

## Abstract

**Background:**

Breast cancer is one of the commonest sources of ocular metastasis. Patients with ocular metastatic disease can present with a variable clinical picture. Patients with a history of breast cancer presenting with any eye symptom should be evaluated with consideration of ocular metastasis.

**Case presentation:**

We report a case of ocular metastasis in a 46-year-old Brahmin woman presenting with right eye pain. She had been treated for stage IIIc left-sided breast cancer 2 years ago with six cycles of chemotherapy with docetaxel, adriamycin, and cyclophosphamide after undergoing modified radical mastectomy. An ophthalmic examination revealed a tender subconjunctival swelling superotemporally on retracting right upper eyelid. This finding alone indicated anterior scleritis. On examining fundus under mydriasis, an amelanotic subretinal mass could be visualized in the posterior pole superotemporal to macula. An orbital magnetic resonance imaging revealed a mass of 2 × 1 cm in size in the subretinal space of her right eye. Computed tomography of her chest was then done and showed multiple metastases in both lungs.

**Conclusion:**

This case report highlights the fact that any unusual ocular presentation, even one simulating anterior scleritis, in a patient with a history of breast cancer should raise suspicion of metastasis.

## Background

Ophthalmic involvement can occur in up to one-fourth of patients with known metastatic breast carcinoma [1]. Although almost any structure of the eye and orbit can be the site for metastasis, the most favored site is the choroid; the choroid is predominantly affected with an incidence of nearly 80% of total ocular metastasis [2].

As survival of patients with breast carcinoma prolongs, the incidence of ocular metastasis and range of ocular morbidity is bound to increase [3]. Although uncommon, ocular metastasis can represent the initial manifestation of an undiagnosed primary tumor [4]. Hence, ophthalmologists in close association with oncologists can play a vital role in detecting an ocular metastasis from a known primary tumor or a primary tumor when an ophthalmic symptom is the initial manifestation of breast cancer.

Ocular metastasis from breast cancer can mimic a variety of conditions depending on the site of metastasis [3]. Here we report a rare case of choroidal metastasis extending anteriorly causing a tender subconjunctival swelling similar to anterior scleritis. This case report serves as an indication that any signs of anterior scleritis in a patient with underlying breast cancer could be associated with ocular metastasis.

## Case presentation

A 46-year-old Brahmin woman with a history of a hormone receptor-negative invasive ductal type of adenocarcinoma of her left breast (stage pT3N3aM0) presented with pain and blurring of vision of her right eye.

Diagnosis of left-sided stage IIIc breast carcinoma was made 2 years ago when she developed pain in her left arm. She subsequently underwent a left-sided modified radical mastectomy. Postoperatively she started treatment with docetaxel 80 mg, adriamycin 60 mg, and cyclophosphamide 600 mg. After completion of six cycles of chemotherapy, external beam radiation of her thoracic wall was performed once a week for 8 weeks.

**Fig. 1:**
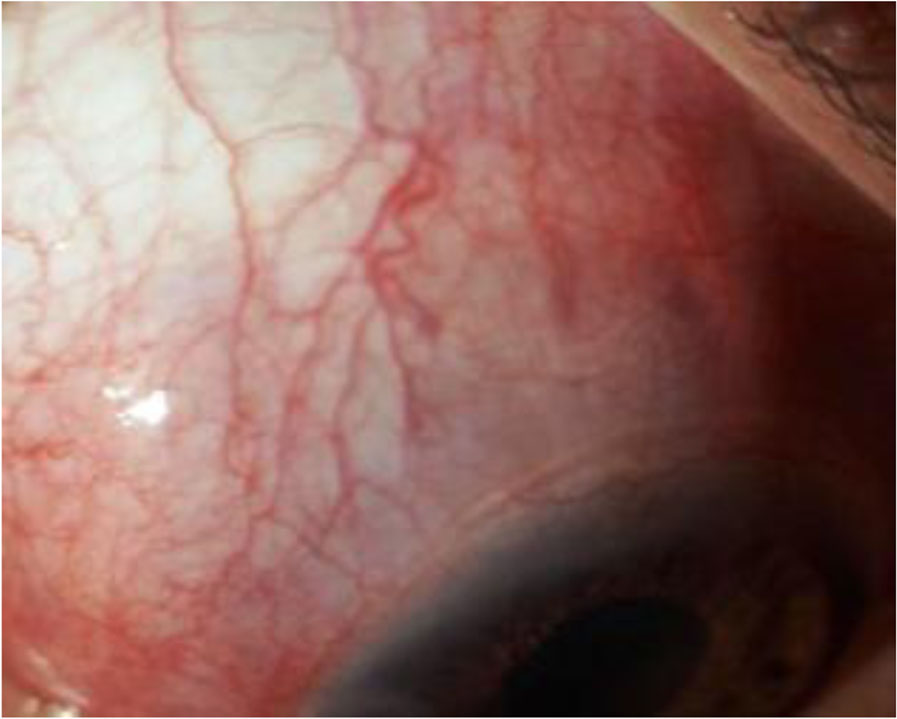
Subconjunctival mass in superotemporal aspect of *right* eye

Two years after initial diagnosis, she presented with temporal headache and pain on temporal aspect of her right eye. Visual acuity in her right eye was “counting fingers” close to face and 6/9 in her left eye. A slit lamp examination revealed a tender subconjunctival swelling with localized congestion in superotemporal aspect of her right eye underneath the upper eyelid (Fig. 1). With this finding alone, an initial clinical diagnosis of anterior scleritis was made until posterior segment was examined. Her pupil was sluggishly reactive with no relative afferent pupillary defect (RAPD).

**Fig. 2:**
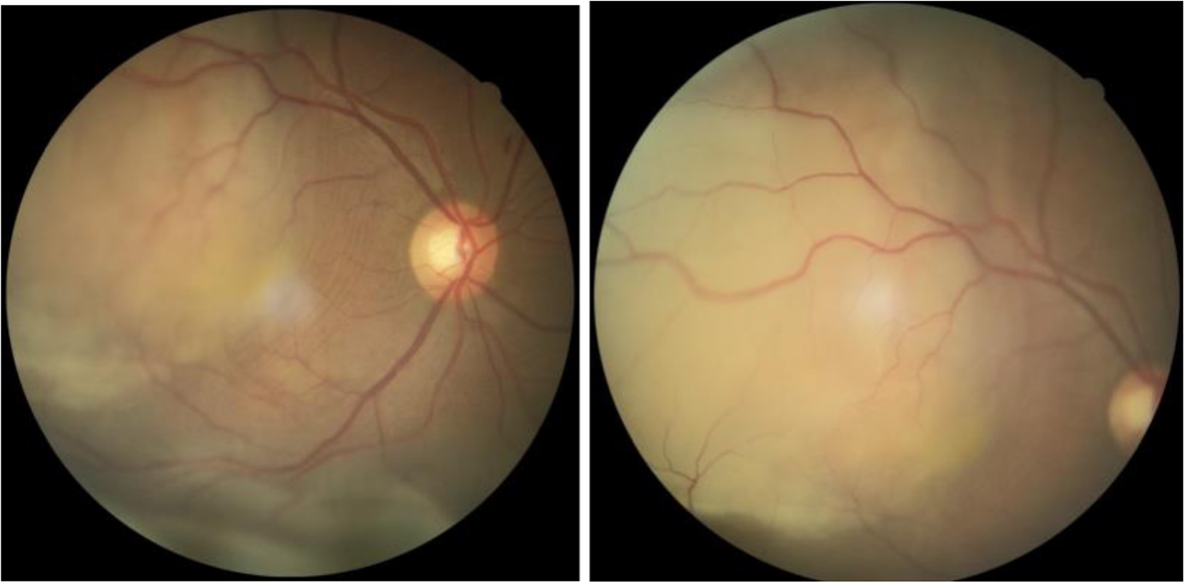
Fundus photographs showing amelanotic subretinal mass in superotemporal quadrant with inferior serous retinal detachment

Fundus evaluation under mydriasis revealed a shallow retinal detachment with underlying amelanotic subretinal mass, superotemporal to macula, causing retinal folds in macular area. A shallow serous inferior retinal detachment could be appreciated inferior to inferotemporal arcade (Fig. 2). Intraocular pressures were normal in both her eyes (10 mm of Hg in right eye and 16 mm of Hg in left eye).

**Fig. 3:**
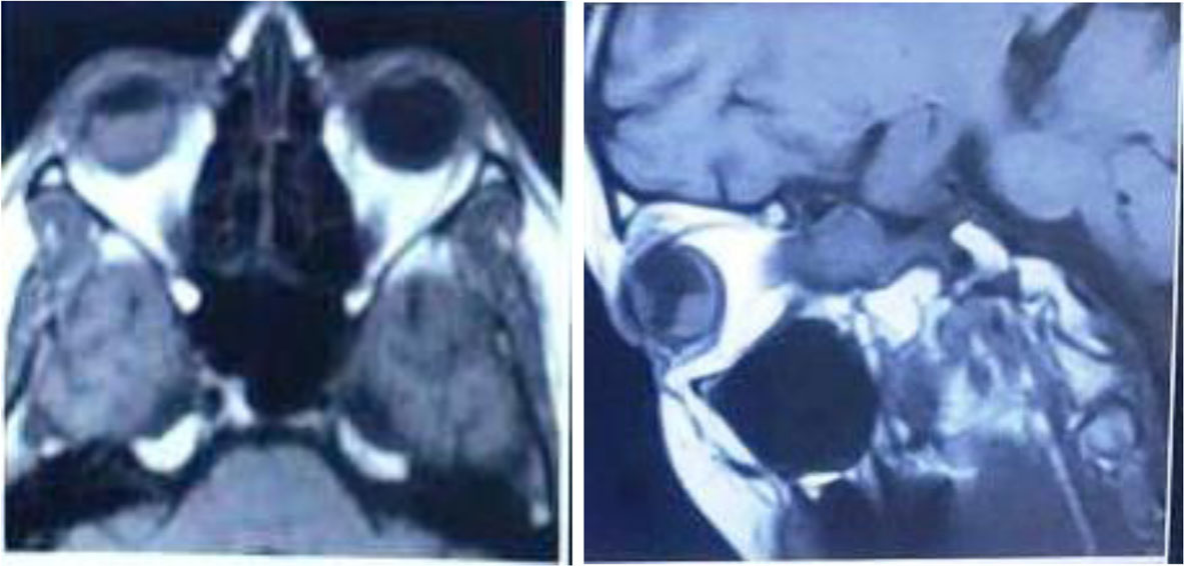
Magnetic resonance imaging orbit and brain showing intraocular mass in *right* eye

Magnetic resonance imaging (MRI) orbit revealed a 2 × 1 cm subretinal mass in superior and temporal aspect of her right eye with enhancement after injection of contrast (Fig. 3). The lesion was of high intensity in T2-weighted images. Brain imaging was normal.

**Fig. 4:**
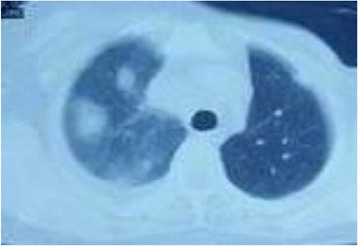
Computed tomography scan of the chest showing pulmonary metastasis

Following this, computed tomography (CT) of her chest was done which showed metastasis in both lungs, mediastinal lymphadenopathy, and right-sided pleural effusion (Fig. 4).

## Discussion

In females, breast cancer is the most common primary tumor and it is responsible for nearly half of the occurrences of ocular metastasis [5]. The uveal tissue, especially the choroid, is the primary ocular site of breast cancer metastases accounting for 81% of total ocular metastasis [2]. The possible explanation for such high metastatic dissemination to the choroid could be its high vascularity [6]. Although most cases of choroidal metastasis are asymptomatic, the most common symptom on presentation is diminution of vision with or without metamorphopsia which can be either due to serous detachment of fovea or macular involvement by malignant lesion [7, 8].

The intraocular anatomical distribution of tumors arising from breast cancer involves mainly the superotemporal quadrant of the fundus [8]. Choroidal foci of metastases typically appear as a homogenous, creamy yellow plateau-shaped lesion located between macula and equator [9], but they can occasionally present with choroidal detachment simulating uveal effusion syndrome or, when advanced, can cause exophthalmos, glaucoma, and uveitis [10]. One unusual reported case presented as scleritis with exudative retinal detachment in a 36-year-old white man; he was diagnosed as having ocular metastasis when enucleation was done to demonstrate small cell carcinoma on histopathological examination after there was no response to treatment and he developed a painful blind eye [11].

Since the present case was a known case of breast carcinoma under routine follow-ups for the last 2 years, diagnosis was much easier. Ocular metastasis usually presents in patients with breast cancer 20 to 40 months after initial diagnosis [12]. Diagnosis of ocular metastatic disease is mainly clinical. When in doubt, investigative modalities such as optical coherence tomography (OCT), fundus fluorescein angiography (FFA), B-scan, and MRI can be helpful. At the time of diagnosis of ocular metastasis, 85% of patients already have lung metastasis [12]. Hence treatment of ocular metastasis is almost always palliative. Prognosis of patients with metastatic ocular tumor is rather poor with a median survival of 9 months [1]. External beam radiation therapy is very effective in patients with blurred vision secondary to foveal involvement and metastatic tumor regression has been reported in 63 to 83% of cases [7, 13].

## Conclusions

Breast cancer is a common source of ocular metastasis. Any ocular features, no matter how subtle or unusual, should raise a suspicion of tumor spread to the eye. As in this case, ocular metastasis can be the first sign of tumor dissemination and thus can aid in further evaluation of systemic involvement.
